# Large enhancement of superconducting transition temperature in single-element superconducting rhenium by shear strain

**DOI:** 10.1038/srep36337

**Published:** 2016-11-04

**Authors:** Masaki Mito, Hideaki Matsui, Kazuki Tsuruta, Tomiko Yamaguchi, Kazuma Nakamura, Hiroyuki Deguchi, Naoki Shirakawa, Hiroki Adachi, Tohru Yamasaki, Hideaki Iwaoka, Yoshifumi Ikoma, Zenji Horita

**Affiliations:** 1 Faculty of Engineering, Kyushu Institute of Technology, Kitakyushu 804-8550, Japan; 2 Flexible Electronics Research Center (FLEC), National Institute of Advanced Industrial Science and Technology (AIST), Tsukuba 305-8565, Japan; 3 Graduate School of Engineering, University of Hyogo, Himeji 671-2280, Japan; 4 Department of Materials Science and Engineering, Faculty of Engineering, Kyushu University, Fukuoka, 819-0395 Japan; 5 International Institute for Carbon-Neutral Energy Research (WPI-I2CNER), Kyushu University, Fukuoka, 819-0395 Japan

## Abstract

Finding a physical approach for increasing the superconducting transition temperature (*T*_c_) is a challenge in the field of material science. Shear strain effects on the superconductivity of rhenium were investigated using magnetic measurements, X-ray diffraction, transmission electron microscopy, and first-principles calculations. A large shear strain reduces the grain size and simultaneously expands the unit cells, resulting in an increase in *T*_c_. Here we show that this shear strain approach is a new method for enhancing *T*_c_ and differs from that using hydrostatic strain. The enhancement of *T*_c_ is explained by an increase in net electron–electron coupling rather than a change in the density of states near the Fermi level. The shear strain effect in rhenium could be a successful example of manipulating Bardeen–Cooper–Schrieffer-type Cooper pairing, in which the unit cell volumes are indeed a key parameter.

Single-element superconductors are Bardeen–Cooper–Schrieffer (BCS)-type superconductors, and volume shrinkage under applied pressure (*P*) is known to be an effective approach to manipulating the superconducting transition temperature (*T*_c_)^1^. When the superconductors are subjected to high pressure, important changes occur in both the conduction electron state and lattice vibration. The change in *T*_c_ under hydrostatic contraction, in which the strain tensor has only diagonal components, is not uniform; in simple metals such as Al, Zn, Ga, Cd, In, Sn, Hg, and Pb, *T*_c_ decreases under pressure, whereas in those such as V, Zr, and Tl, *T*_c_ increases under pressure^1^,^2^. According to the McMillan–Allen–Dynes formula, *T*_c_ can be expressed on a quantitative basis as


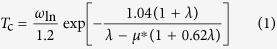


where *ω*_ln_ is the phonon frequency, *λ* is the electron lattice coupling, and μ* is the effective Coulomb potential^3^,^4^. However, the change in *T*_c_ under pressure in simple metals can be understood more concretely using the following formula,


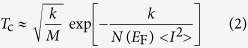


where *M* is the mass of the ions, *k* is the spring constant, *N*(*E*_F_) is the electronic-density of states at the Fermi level *E*_F_, and <*I*^2^> is the average square electronic matrix element^1^,^2^. The product of *N*(*E*_F_) and <*I*^2^> is an electronic term called as the Hopfield parameter *η*^5^. In equation (2), any change in the pre-factor is negligible compared to the terms in the exponent. The relative magnitudes of the increases in *k* and *η* determine whether *T*_c_ will increase or decrease under pressure. Thus, the decrease in *T*_c_ with pressure in simple metals originates mainly from the increase in *k*, *i.e*., lattice stiffening. Thus, in simple metals with d *T*_c_/d*P* < 0, the hydrostatic strain effects are understood to be governed by stiffening in the lattice vibration spectrum rather than the changes in the electronic properties such as broadening of the density of states near the Fermi level^1^,^2^. Here, the electron-lattice coupling parameter *λ* in equation (1) is expressed as *λ* = *N*(*E*_F_) <*I*^2^>/*M* <*ω*^2^>, where <*ω*^2^> is the average square phonon frequency. Further, it is also thought that the decrease in *T*_c_ with pressure in simple metals results from a weakening of *λ* due to the shift of the phonon spectrum to higher frequencies^1^. Interestingly, the hydrostatic pressure effect in rhenium (Re) is unique in that the *T*_c_ exhibits a decrease followed by an increase with a minimum at approximately 0.6 GPa^6^,^7^.

In high-pressure studies, the exploitation of the isotropic features of stress, i.e., hydrostatic pressure, has been viewed as an ideal structural manipulation; the intrinsic response to stress has been studied, and first-principles calculations have been performed. As mentioned above, under hydrostatic pressure conditions, only diagonal components exist in the strain tensor. Indeed, hydrostatic pressure is especially important in the development of organic superconductors, in which nondiagonal components are detrimental to the strain-induced superconductivity^8^. However, in this study of Re, we present a new approach for greatly increasing *T*_c_, in which shear stress instead of hydrostatic pressure is a key parameter affecting the *T*_c_ value of superconductors.

Previous studies of the strain effects in Re are briefly reviewed here. According to an earlier study of Re by Hulm and Goodman in 1957, an arc-melted powder without strain has a sharp superconducting transition at *T*_c_ = 1.70 K^9^. The Meissner effect reveals the magnetic-field-dependent characteristics of typical type-I superconductors. They also reported that Re ground to a cylinder has a *T*_c_ value of 2.7 K, suggesting that some shear strain effect could work positively to increase *T*_c_. However, when pure Re crystals with *T*_c_ of 1.69–1.70 K are placed under hydrostatic pressure, *T*_c_ decreases with a slope of d*T*_c_/d*P* ~ –2 × 10^–2^ K/GPa for *P* < 0.4 GPa, and the initial slope corresponds to approximately −1 × 10^–2^ K at a volume shrinkage of 0.1%^6^,^7^. The change in *T*_c_ is found to increase at around 0.6 GPa, where *T*_c_ has the decrease of approximately 0.01 K. For *P* > 1.6 GPa, the increase in *T*_c_ tends to saturate at *T*_c_ ~ 1.69 K. Thus, the effect of shear strain in Re is completely different from the hydrostatic pressure effects described above, but is instead qualitatively consistent with the effects of doping with Os or W^10^. Generally, the shear strain reduces the domain size; hence it is not considered a promising factor for physical manipulation. Here, we consider the shear strain effect in Re using the proposed new approach.

The experimental apparatus for our high-pressure experiments was a diamond anvil cell^11^. A gasket was clamped between two diamond anvils. The gasket material must be hard and resistant to any stress. In fact, Re is often used as a gasket material. From our experience, we have found that a Re gasket often has a *T*_c_ above 2.0 K, and *T*_c_ depends on the degree of prior treatment (that is, it is related to the magnitude of internal strain in the gasket). Indeed, this phenomenon is the same as the strain effect reported by Hulm and Goodman^9^. Given the above background, we systematically study the effect of shear strain on the superconductivity of Re over a wide strain range.

Severe plastic deformation can be used to apply a large strain to materials, resulting in bulk nanostructured materials with ultrafine-grained structures^12^. Examples of such deformation include equal-channel angular pressing^13^, accumulative roll-bonding^14^, and high-pressure torsion (HPT)^15^. In 1935, Bridgman introduced the HPT method combined with hydrostatic pressure as a method for obtaining high shearing stress^16^. In particular, this HPT method can introduce intense shear strain so that the materials have ultrafine grains on the submicrometer or nanometer scale^15^,^17^,^18^,^19^. In the present study, the HPT method is adopted to apply intense shear strain to Re. The shear strain effects are physically elucidated using structural analysis and first-principles calculations.

## Results

### Magnetic measurements

**Figure 1:**
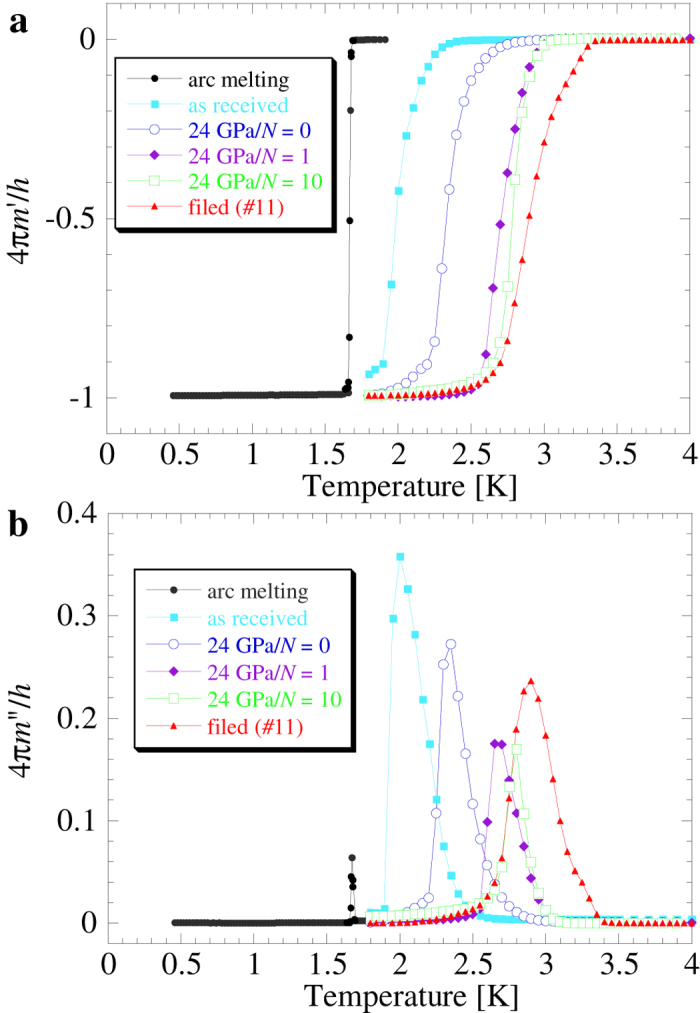
Temperature dependence of the (**a**) in-phase ac susceptibility 4π*m*’/*h* and (**b**) out-of-phase one 4π*m*”/*h* for Re processed by HPT at 24 GPa with *N* = 0, 1, and 10. The in-phase susceptibility is shown in SI units in order to evaluate the volume fraction. Data for the arc-melted and as-received samples are also shown for reference.

**Figure 2:**
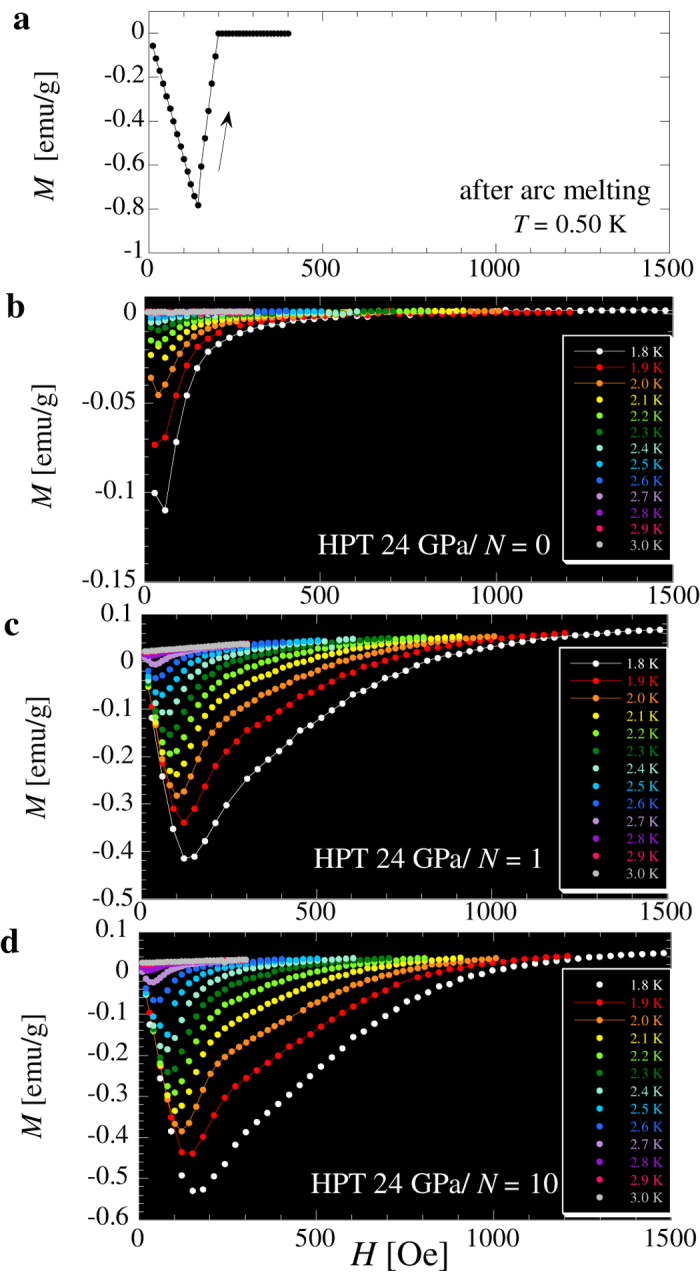
(**a**) Magnetization *M* as a function of dc magnetic field (*H*) for the arc-melted sample. (**b**–**d**) Temperature dependence of *M* for HPT-treated Re for (**b**) *N* = 0, (**c**) *N* = 1, and (**d**) *N* = 10. *M* is evaluated using CGS units in order to extract the shift of the Meissner signal.

The in-phase and out-of-phase ac magnetic susceptibility (denoted by 4π*m*’/*h* and 4π*m*”/*h*, respectively) as a function of temperature (*T*) for the arc-melted sample are shown in Fig. 1(a,b), respectively, and Fig. 2(a) shows the magnetization (*M*) as a function of the dc magnetic field (*H*). At around 1.7 K, a sharp Meissner signal appeared within a temperature width of 20 mK, and *T*_c_ was estimated to be 1.7 K. We confirmed that in a spherical sample of arc-melted material, an almost perfect Meissner signal appeared. In the disk samples, a Meissner signal with a similar magnitude was observed. The magnetization *M*(*H*) exhibited a sharp jump at around *H* = 170 ± 30 Oe, indicating that the unstrained sample was a type-I superconductor. The results are consistent with those reported by Hulm and Goodman^9^.

Figure 1 also shows the temperature dependence of the in-phase and the out-of-phase ac susceptibility for HPT-processed Re. In the 4π*m*’/*h* data, the Meissner signal shifted toward the high-temperature side with increasing strain. When we evaluated *T*_c_ according to the onset of 4π*m*’/*h*, *T*_c_ was estimated to be 2.5, 2.9, and 3.0 K for *N* = 0, 1, and 10, respectively, where *N* is the number of revolutions in the HPT process. For reference, the *T*_c_ value of the as-received sample was 2.2 K. We recognize a large enhancement of *T*_c_ caused by torsion. The susceptibility 4π*m*”/*h* reveals the energy loss against the ac magnetic field and the distribution of *T*_c_. The peak of 4π*m*”/*h* corresponds to the midpoint of a large decrease in 4π*m*’/*h*. The upper limit of a finite *m*” corresponds to a possible optimal *T*_c_. Even at *N* = 0, the optimal *T*_c_ of about 2.8 K implies the possibility of increasing *T*_c_. The possible *T*_c_ for *N* = 10 is 3.2 K. We confirmed that the possible *T*_c_ of Re was enhanced to 3.4 K when the sample was filed using sandpapers as shown in Fig. S4, suggesting the importance of using shear strain instead of hydrostatic compression.

Figure 2(b–d) shows *M*(*H*) for the HPT-processed Re at *T* < *T*_c_. The field region of the Meissner signal was greatly enhanced with increasing *N*, and the type of superconductivity, determined by extracting the magnetic flux, changed from type-I to type-II. At *T* = 1.8 K, the lower critical field *H*_c1_ is less than 30, 90, and 130 Oe for *N* = 0, 1, and 10, respectively. Furthermore, the upper critical field *H*_c2_ at *T* = 1.8 K is 0.7, 1.3, and >1.5 kOe for *N* = 0, 1, and 10, respectively. Thus, a reduction in the coherence length *ξ* was experimentally suggested by the change in the magnetic field dependence of the Meissner signal as well as the change in *T*_c_ (∝*ξ*^−1^). The positive effect of the shear strain appears in the increases in *H*_c1_ and *H*_c2_ as well as *T*_c_.

### Structural characterization

**Figure 3:**
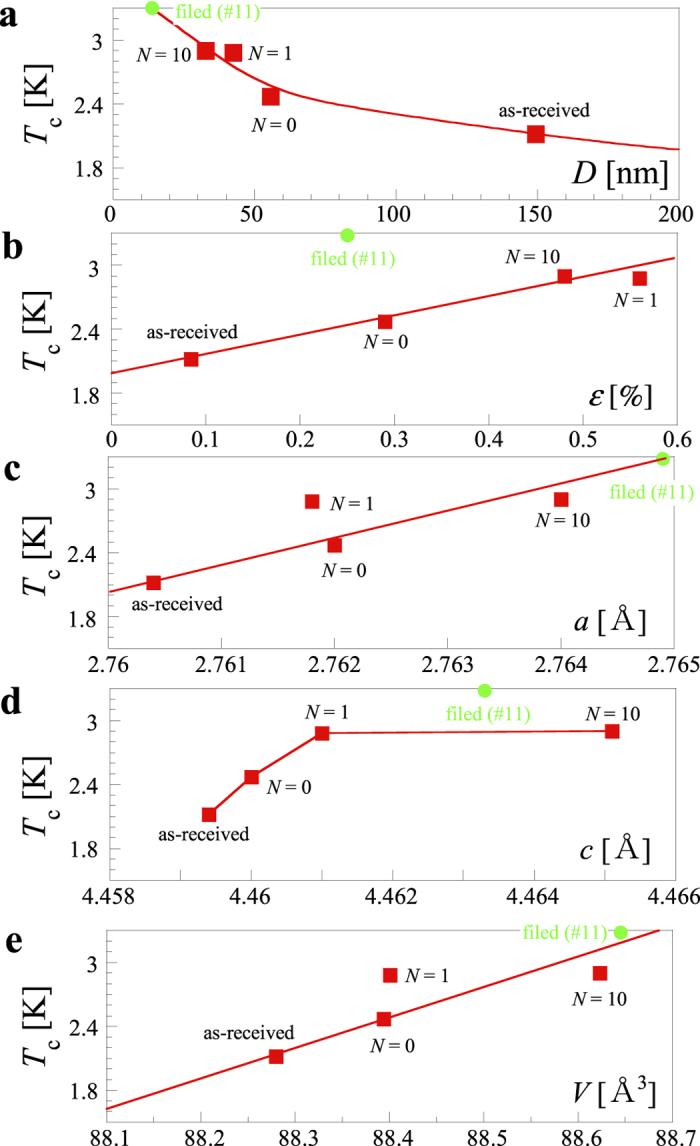
Relation between *T*_c_ and the following structural parameters; (**a**) crystalline size *D*, (**b**) crystalline strain *ε*, the lattice parameters (**c**) *a*, (**d**) *c*, and (**e**) unit cell volume *V*. A series of experimental data was obtained for the as-received, HPT-treated, and filed specimens.

Figure S2 shows the X-ray diffraction profiles of the as-received and HPT-processed samples. The shift in a series of diffraction peaks yields information about the lattice parameters. The half**-**width of the diffraction peaks indicates the average grain size and internal strain. Figure 3 shows the relation between *T*_c_ and several structural parameters; the crystalline size *D* (a), crystalline strain *ε* (b), lattice parameters *a* (c) and *c* (d), and unit cell volume *V* (e). These figures include data for the as-received specimen, the HPT-processed ones (*N* = 0, 1, and 10 at *P* = 24 GPa), and a filed specimen (#11 in Table S1). The filing was performed under a slight stress along the direction perpendicular to the disk, and the sample was subjected to strong strain caused by rotation. The shear strain accompanying rotation on the *c*-plane was thought to expand the size of the *c*-plane and decrease the crystalline size. As the shear strain increased, expansion along the *a*-axis also occurred, resulting in an increase in the unit cell volume. Indeed, the largest expansion along the *a*-axis was observed in the filed Re rather than the HPT-processed Re with *N* = 10. According to the results for the as-received, HPT-processed, and filed specimens, *D*, *a*, and *V* are promising parameters for scaling *T*_c_. Figure S3 shows a transmission electron microscopy micrograph of the sample processed by HPT at *P* = 24 GPa and *N* = 10. The grain size was reduced to the order of ~100 nm, suggesting a reduction in the coherence length. As shown in Fig. 1, a prominent Meissner signal was observed, and the present materials were considered to be strongly coupled superconducting-grain systems.

### Band structure calculations

**Figure 4:**
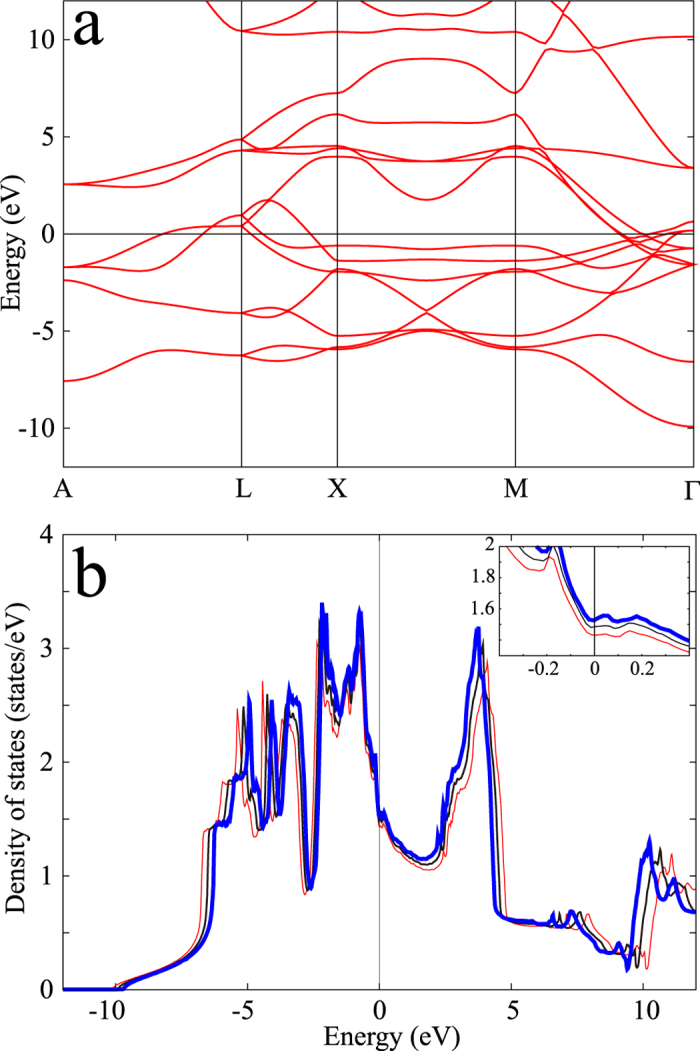
(**a**) Density-functional band structure calculated for an experimental Re structure with *a* = 2.758 Å and *c* = 4.447 Å. (**b**) Effect of the lattice change on the density of states, where we scale the lattice parameters by the factor *α*. Thin-red, black, and thick-blue curves indicate the results for *α* = 0.99, 1, and 1.01, respectively. The results near the Fermi level (*E* = 0) are enlarged in the inset. An overall shrinkage of the density of states toward the Fermi level (*E* = 0) was observed because of a lattice expansion of 1%. The Fermi surfaces for *α* = 0.99, 1.00, and 1.01 are shown in Fig. S5.

To see the effect of changes in the electronic structures, we performed band structure calculations for Re with different configurations. Figure 4(a) shows the calculated ab-initio band structure for an experimental structure with *a* = 2.758 Å and *c* = 4.447 Å, and Fig. 4(b) compares the densities of states for different lattice parameters. A slight lattice expansion (around 1%) gave rise to appreciable overall shrinkage of the density of states toward to the Fermi level (*E* = 0) (thin red to thick blue curves). In particular, a shift of the peak around 5 eV was noticeable. These observations indicate a lowering of the excitation energy caused by volume expansion. In many-body perturbation theory^20^,^21^,^22^, the lowering of the excitation energy is generally associated with enhancement of the polarization function. This polarization enhancement, i.e., the large dielectric screening, can weaken the effective repulsion between electrons. We thus expect the electronic pseudopotential *μ* and also μ* to reduce with volume expansion. In addition, we see in the inset in panel (b) that the density of states at the Fermi level increases with volume expansion. In 5d metals, the increase in the density of states at the Fermi level can generally lead to enhancement of the electron-phonon coupling *λ*^3^. The change in these parameters supports reasonably well the presented experimental observation that volume expansion (i.e., the shear strain) leads to an enhancement of *T*_c_. We note that the same tendency was observed in alkali-doped C_60_ systems^23^. Moreover, it is known that Re uniquely exhibits an increase in *T*_c_ (at maximum, 2.2 K) when some Re is replaced with Os or W, in which the electronic density of states increases just below the Fermi level^10^. According to Chu *et al*., the change in the *T*_c_ value of Re with alloying can be understood in terms of a change in *λ*^10^. The present results are reasonably consistent with the effect of replacing some Re with Os or W, where the former exhibits a more drastic increase in *T*_c_.

## Discussion

Here we discuss some factors that could be promising for increasing *T*_c_. According to the BCS theory, *T*_c_ increases as the density of states at the Fermi level increases, because the number of electron pairs contributing to the superconductivity increases there. Furthermore, according to the MAD formula in equation (1), *T*_c_ increases as the phonon frequency becomes higher and the electron-phonon interaction becomes larger. In previous high-pressure experiments on single-element superconductor**s**, much attention was given to changes in the phonon stiffening as well as the density of states. In the present work, the most important factor is the electron-phonon interaction. This tendency is unique in a series of high-strain experiments on single-element superconductor**s**, suggesting a new approach to modifying *T*_c_. Under hydrostatic pressure, *T*_c_ decreases with a slope of approximately −1 × 10^–2^ K for a volume shrinkage of 0.1%^6^,^7^. However, in the HPT process including shear strain, *T*_c_ increases with a slope of 1.5 K for a volume expansion of 0.7%, which is more than 20 times the change rate under hydrostatic pressure^6^,^7^. Thus, the effect of shear strain is expected to be a promising factor in enhancing *T*_c_.

In conclusion, Re subjected to HPT has an ultrafine structure in which the grain size decreases to below the submicrometer level and the lattice expands. The superconducting transition temperature *T*_c_ increases with increasing strain, and an important strain component here is shear instead of hydrostatic compression. According to first-principles calculations, the density of states at the Fermi energy increases only slightly, but the van-Hove-singularity shifts toward the low-energy side, suggesting enhanced screening. This means that electrons are affected by fluctuation of the lattice, resulting in an increase in the electron-phonon coupling. Thus, a change in the electron-phonon coupling instead of the density of states at the Fermi level gives rise to an increase in *T*_c_.

## Methods

### Materials

High-purity Re disks (99.97%, Johnson Mathey) with 4 mm in diameter and 0.25 mm in thickness were subjected to HPT processing under a pressure of *P* = 24 GPa for the revolution number of HPT process *N* = 0, 1, and 10 turns, as shown in Fig. S1. Tool steel was used for the anvils for *N* = 0 and 1, and tungsten carbide was used for *N* = 10. The pressure of 24 GPa is the value given by the applied load, 50 tons, divided by the total area of the central shallow hole made on the anvils, (2.5 mm)^2^π. This pressure may be slightly overestimated if a burr is formed during the processing. When we used tungsten carbide anvils, processing was feasible without breaking them. Note that not only the strength of the anvils but also the geometry is important for maintaining feasible processing. The strain imposed on the sample was estimated by *ε* = 2π *r N* /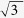
*t* (where *r* is the radius and *t* is the thickness) as the equivalent strain^24^,^25^. The shear strain generated by HPT processing typically decreases the grain size and thus increases the grain boundary area. As a reference sample, a ball-like sample, whose residual strain was removed, was prepared by sufficient arc melting. Furthermore, for another reference, a Re plate was filed by sandpapers to introduce random shear strain under a small stress perpendicular to the disk. The number of times the plate was filed is denoted by *n* (the detailed experimental data are shown in the supplemental material). Indeed, the HPT effect has already been studied for other single-element superconductors; a type-II superconductor, niobium (Nb), with d*T*_c_/d*P* = –16 to –25 mK/GPa at low pressures, has a maximum *T*_c_ of approximately 10 K at around 10 GPa^26^. However, Nb prepared by HPT (6 GPa) exhibits an increase in *T*_c_ of only ~0.10 K at maximum^27^. On the other hand, a thin film with a 100 nm thickness under hydrostatic pressure exhibits an increase in *T*_c_ with d*T*_c_/d*P* = 73 mK/GPa even at a low pressure^28^. The HPT effect in Nb is qualitatively consistent with the hydrostatic pressure effect in a Nb film. Given the above background, the HPT effect in Re with *T*_c_ > 1.5 K reported herein is relatively large; hence, we need to understand the effects of the nondiagonal as well as diagonal components of the strain tensor.

### Magnetic measurements

The superconducting transition was investigated by observing the Meissner signal in the ac magnetic susceptibility using a superconducting quantum interference device magnetometer (Quantum Design Inc.) equipped with an ac measurement option and a ^3^He refrigerator option (IQUANTUM)^29^. The frequency and amplitude (*h*) of the ac magnetic field were 10 Hz and 4 Oe, respectively. In the ac magnetic susceptibility measurements, the data for the magnetization *M* were gathered as a function of time *t*. Fourier analysis of the *M*(*t*) data yields the amplitudes of both the in-phase component (*m*’) and that of the out-of-component (*m*”). Both *m*’ and *m*” are divided by the amplitude of the ac field (*h*) to obtain the ac magnetic susceptibility. For the spherical sample of arc-melted material, the demagnetization field effect was calibrated using a demagnetization coefficient of 1/3, and we obtained a value of 99.4% as the volume fraction of the superconductor. To reduce the diamagnetic effect, the disks of the HPT**-**treated and filed specimens were placed so that the normal vectors of their round surfaces were perpendicular to the dc magnetic field. The stability of superconductivity in a magnetic field was investigated using the dc magnetic measurement.

### X-ray diffraction (XRD)

The grain size, strain, and unit cell parameters were investigated in XRD experiments using an X-ray diffractometer (Rigaku, SmartLab) with Cu-Kα radiation at 45 kV and 200 mA. The grain size and strain were calculated from the integrated intensities of the diffracted peaks using Williamson-Hall analysis^30^.

### Transmission electron microscopy (TEM)

Microstructural observation was performed using TEM, and the grain size was measured using dark-field images. The grain size estimated by XRD is generally smaller than that measured by TEM.

### Band calculation

Density-functional band structure calculations were performed for several lattice configurations of Re to study the structural change effect on the low-energy electronic structures. Then, first-principles calculations were performed using the Tokyo Ab-initio Program Package^31^ with plane-wave basis sets, where norm-conserving pseudopotentials^32^,^33^ and the generalized gradient approximation of the exchange-correlation potential^34^ with partial core correction were employed. The cutoff energies for the wave function and charge densities are 49 and 196 Ry, respectively, and 41 × 41 × 41 k-point sampling was employed. The integral over the Brillouin zone was evaluated by the tetrahedron method with a broadening of 0.01 eV. The density of states for Re at the Fermi energy (*E* = 0) is estimated to be 1.486/eV, which is equivalent to 0.372 (eV·spin·atom)^−1^. This value is consistent with the value of 0.33 (eV·spin·atom) ^−1^ in McMillan’s study^3^. We also calculated the Fermi surface^35^ to see the volume change effect on the electronic structure.

## Additional Information

**How to cite this article**: Mito, M. *et al*. Large enhancement of superconducting transition temperature in single-element superconducting rhenium by shear strain. *Sci. Rep*. **6**, 36337; doi: 10.1038/srep36337 (2016).

**Publisher’s note:** Springer Nature remains neutral with regard to jurisdictional claims in published maps and institutional affiliations.

## Supplementary Material

Supplementary Information

## References

[b1] Schilling J. S. High-Pressure Effects in Handbook of High-Temperature Superconductivity edited by J. R. Schrieffer (Springer, New York, 2007).

[b2] Hamlin J. J. Superconductivity in the metallic elements at high pressures. Physica C 514, 59–76 (2015).

[b3] McMillan W. L. Transition temperature of strong-coupled superconductors. Phys. Rev. 167, 331–344 (1968).

[b4] Allen P. B. & Dynes R. C. Transition temperature of strong-coupled superconductors reanalyzed. Phys. Rev. B12, 905–922 (1975).

[b5] Hopfield J. J. On the systematics of high *T*_c_ in transition metal materials. Physica 55, 41–49 (1971).

[b6] Chu C. W., Smith T. F. & Gardner W. E. Superconductivity of rhenium and some rhenium-osmium alloy s at high pressure. Phys. Rev. Lett. 20, 198–201 (1968).

[b7] Chu C. W., Smith T. F. & Gardner W. E. Study of Fermi-surface topology changes in rhenium and dilute Re solid solutions from *T*_c_ measurements at high pressure. Phys. Rev. B1, 214–221 (1970).

[b8] Campos C. E. . Uniaxial-stress-induced superconductivity in organic conductors. Phys. Rev. B52, R7014–R7017 (1995). 10.1103/physrevb.52.r7014 9979738

[b9] Hulm J. K. & Goodman B. B. Superconducting Properties of Rhenium, Ruthenium, and Osmium. Phys. Rev. 106, 659–671 (1957).

[b10] Chu C. W., McMillan W. L. & Luo H. L. Superconductivity of Re-Os, Re-Ru, Ru-Os, and Re-W hcp alloy systems and slightly doped Re. Phys. Rev. B3, 3757–3762 (1971).

[b11] Eremets M. High Pressure Experimental Methods (Oxford University Press, New York, 1996).

[b12] Valiev R. Z. . Producing Bulk Ultrafine-Grained Materials by Severe Plastic Deformation. JOM 58, 33–39 (2006).

[b13] Segal V. M., Drobyshevskii V. I. A. E. & Kopylov V. I. Plastic working of metals by simple shear. Russian Metal 1, 99–105 (1981).

[b14] Saito Y., Utsunomiya H., Tsuji N. & Sakai T. Novel ultra-high straining process for bulk materials-development of the accumulative roll-bonding (ARB) process. Acta Mater. 47, 579–583 (1999).

[b15] Smirnova N. A. . Evolution of the structure of f.c.c.single crystal subjected to strong plastic deformation. Fiz. Metal. Metalloved. 61, 1170–1177 (1986).

[b16] Bridgman P. W. Effects of High Shearing Stress Combined with High Hydrostatic Pressure. Phys. Rev. 48, 825–847 (1935).

[b17] Harai Y., Ito Y. & Horita Z. High-pressure torsion using ring specimens. Scripta Mater. 58, 469–472 (2008).

[b18] Edalati K. & Horita Z. High-pressure torsion of pure metals: Influence of atomic bond parameters and stacking fault energy on grain size and correlation with hardness. Acta Mater. 59, 6831–6836 (2011).

[b19] Edalati K. & Horita Z. Correlations between hardness and atomic bond parameters of pure metals and semi-metals after processing by high-pressure torsion. Scripta Mater. 64, 161–164 (2011).

[b20] Nakamura K., Arita R. & Imada M. Ab initio Derivation of Low-Energy Model for Iron-Based Superconductors LaFeAsO and LaFePO. J. Phys. Soc. Jpn. 77, 093771 (2008).

[b21] Nakamura K., Nohara Y., Yoshimoto Y. & Nomura Y. Ab initio GW plus cumulant calculation for isolated band system: Application to organic conductor (TMTSF)_2_PF_6_ and transition-metal oxide SrVO_3_. Phys. Rev. B 93, 085124 (2016).

[b22] Akashi R., Nakamura K., Arita R. & Imada M. High-temperature superconductivity in layered nitrides *β*-Li*xM*NCl (*M* = Ti, Zr, Hf): Insights from density functional theory for superconductors. Phys. Rev. B 86, 054513 (2012).

[b23] Ganin A. Y. . Bulk superconductivity at 38K in a molecular system. Nature Materials 7, 367–371 (2008). 18425134 10.1038/nmat2179

[b24] Wetscher F., Vorhauer A., Stock R. & Pippan R. Structural refinement of low alloyed steels during severe plastic deformation. Mater. Sci. Eng. A387–389, 809–816 (2004).

[b25] Hebesberger T., Stüwe H. P., Vorhauer A., Wetcher F. & Pippan R. Structure of Cu deformed by high pressure torsion. Acta Mater. 53, 393–402 (2005).

[b26] Smith T. F. Pressure dependence of the superconducting transition for niobium. Phys. Lett. A 33, 465–466 (1970).

[b27] Nishizaki T., Lee S., Horita Z., Sasaki T. & Kobayashi N. Superconducting properties in bulk nanostructured niobium prepared by high-pressure torsion. Physica C 493, 132–135 (2013).

[b28] Pristáš G. . Influence of hydrostatic pressure on superconducting properties of niobium thin film. Thin Solid Films 556, 470–474 (2014).

[b29] Shirakawa N. & Tamura M. Low temperature static magnetization of an organic ferromagnet, β-p-NPNN. Polyhedron 24, 2405–2408 (2005).

[b30] Williamson G. K. & Smallman R. E. Dislocation densities in some annealed and cold-worked metals from measurements on the X-ray debye-scherrer spectrum. Philos. Mag. 1, 34–46 (1956).

[b31] Yamauchi J., Tsukada M., Watanabe S. & Sugino O. First-principles study on energetics of *c*-BN(001) reconstructed surfaces. Surface Science 341, L1037–L1041 (1995). 10.1103/physrevb.54.5586 9986522

[b32] Troullier N. & Martins J. L. Efficient pseudopotentials for plane-wave calculations. Phys. Rev. B 43, 1993–2006 (1991). 10.1103/physrevb.43.1993 9997467

[b33] Kleinman L. & Bylander D. M. Efficacious Form for Model Pseudopotentials. Phys. Rev. Lett. 48, 1425–1428 (1982).

[b34] Perdew J. P., Burke K. & Ernzerhof M. Generalized Gradient Approximation Made Simple. Phys. Rev. Lett. 77, 3865–3868 (1996). 10062328 10.1103/PhysRevLett.77.3865

[b35] Kawamura M., Gohda Y. & Tsuneyuki S. Improved tetrahedron method for the Brillouin-zone integration applicable to response functions. Phys. Rev. B 89, 094515 (2014).

