# Supplementary material for: Large enhancement of superconducting transition temperature in single-element superconducting rhenium by shear strain
Source: Sci Rep. 2016 Nov 4;6:36337. doi: 10.1038/srep36337 (PMC5095657; doi:10.1038/srep36337)
Supplement: Supplementary Information [file srep36337-s1.doc]

**Supporting Information**

Large enhancement of superconducting transition temperature in single-element superconducting rhenium by shear strain

Masaki Mito^1*^, Hideaki Matsui^1^, Kazuki Tsuruta^1^, Tomiko Yamaguchi^1^, Kazuma Nakamura^1^, Hiroyuki Deguchi^1^, Naoki Shirakawa^2^, Hiroki Adachi^3^, Tohru Yamasaki^3^, Hideaki Iwaoka^4^, Yoshifumi Ikoma^4^, and Zenji Horita^4, 5^

^1^ *Faculty of Engineering, Kyushu Institute of Technology, Kitakyushu 804-8550, Japan*

^2^ *Flexible Electronics Research Center (FLEC), National Institute of Advanced Industrial Science and Technology (AIST), Tsukuba 305-8565, Japan*

^3^ *Graduate School of Engineering, University of Hyogo, Himeji 671-2280, Japan*

^4^ *Department of Materials Science and Engineering, Faculty of Engineering, Kyushu University, Fukuoka, 819-0395 Japan.*

^5^ *International Institute for Carbon-Neutral Energy Research (WPI-I2CNER), Kyushu University, Fukuoka, 819-0395 Japan.*

*Correspondence to mitoh@mns.kyutech.ac.jp

**Table of Contents**

**Fig. S1** Overview of the approach of preparing the studied Re sample. **·································**S3

**Fig. S2** X-ray diffraction (XRD) profiles for as-received, HPT, and filed samples. **···········**S4

**Fig. S3** (**a**) Selected area electron diffraction (SAED) pattern, (**b**) transmission electron microscopy (TEM) bright-field image and (**c**) dark-field image of HPT-processed sample under *P* = 24 GPa for *N* = 10.**······································································································**S5

**Fig. S4** Temperature dependence of the in-phase (*m*') (**a**) and the out-of-phase (*m*'') (**b**) of ac magnetic susceptibility for a series of filed Re specimens. **··················································**S6

**Fig. S5** Calculated Fermi Surface for Re with different lattice parameters. **····························**S7

**Table S1** Physical properties of a series of filed Re specimens. **··········································**S8

**Table S2** Structural parameters such as the crystalline size *D*, crystalline strain *ε*, lattice constants *a*, and *c* in strained Re. **·······················································································**S9


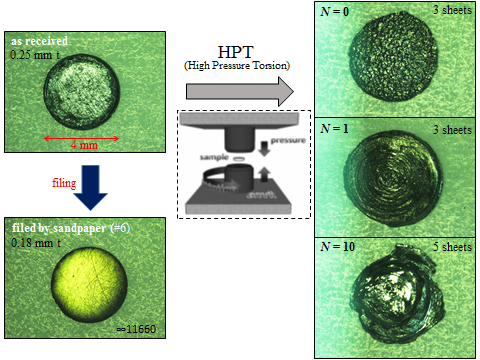


**Fig. S1.**

**Overview of the approach of preparing the studied Re sample.** There are two types of yielding the strain such as the high-pressure torsion (HPT) and the simple filing by sandpapers.

**Fig. S2**

**X-ray diffraction (XRD) profiles for as-received, HPT, and filed samples** (#11 in Tables S1 and S2), obtained by the XRD experiments using X-ray diffractometer (Rigaku, SmartLab) with Cu-Kα radiation at 45 kV and 200 mA. Here are presented the diffraction peaks of the plane index (002), which have the largest diffraction intensity among a series of diffraction peaks. The shift in a series of diffraction peaks yields information of the lattice parameters. The half-width of the diffraction peaks indicates the average grain size and the internal strain. The measurement of the as-received sample has been conducted twice, and the data for as received (2) are presented in Fig. 3.


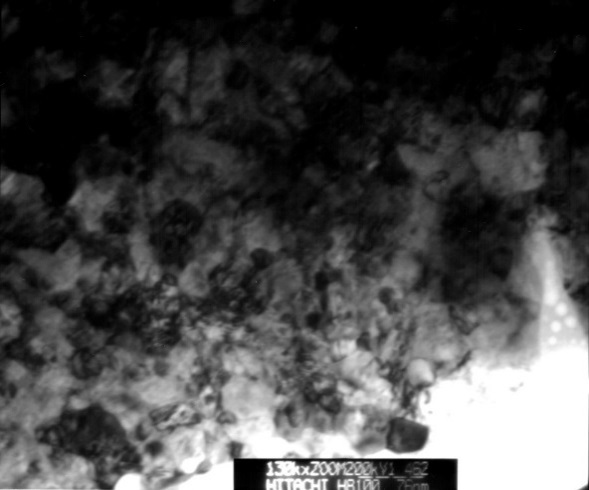

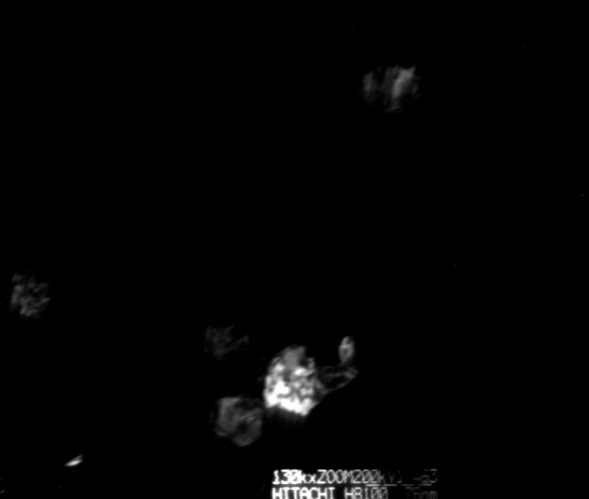


100 nm


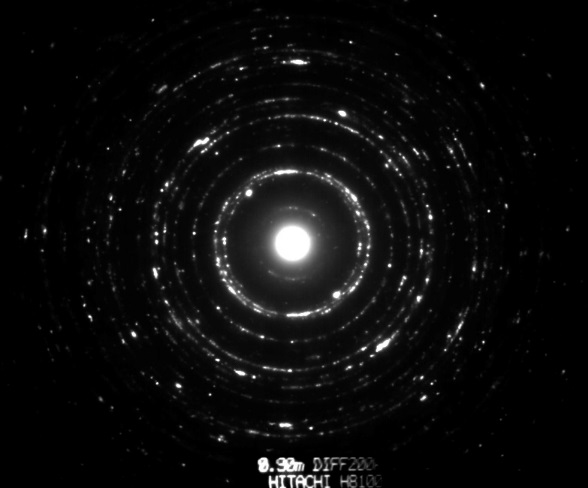


100

002

101

102

103

110

**a**

**b**

**c**

SI-3

**Fig. S3**

**(a) Selected area electron diffraction (SAED) pattern, (b) transmission electron microscopy (TEM) bright-field image and (c) dark-field image of HPT-processed sample under *P* = 24 GPa for *N* = 10.** The SAED pattern was taken from a region of ~1.3 μm in diameter and contains many diffracted beams around rings, indicating the formation of nanograins with high-angles of misorientation. The dark-field image was taken by diffracted beams indicated by an arrow in the SAED pattern. The grain size is of the order of ~100 nm. Generally the grain size estimated by XRD is smaller than that measured by TEM.

**Fig. S4**

**Temperature dependence of the in-phase (*m*') (a) and the out-of-phase (*m*'') (b) of ac magnetic susceptibility for a series of filed Re specimens.** Each magnitude is normalized by the maximum of ac magnetic susceptibility (*m*_s_). The detailed explanation on the specimens is presented in Table S1.

~~
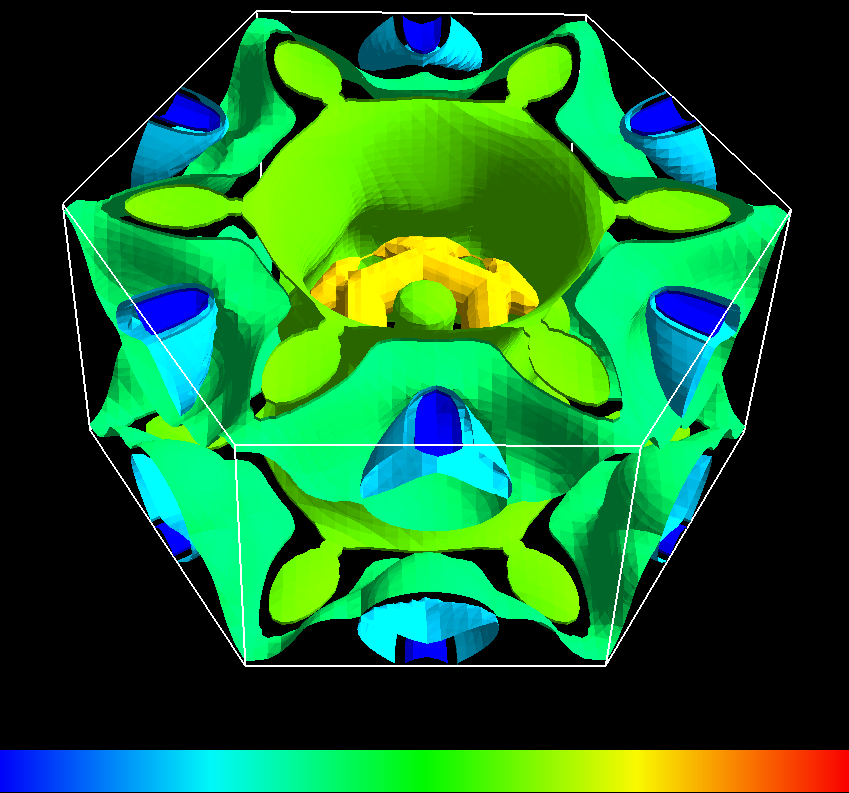
~~

**a**

α = 0.99


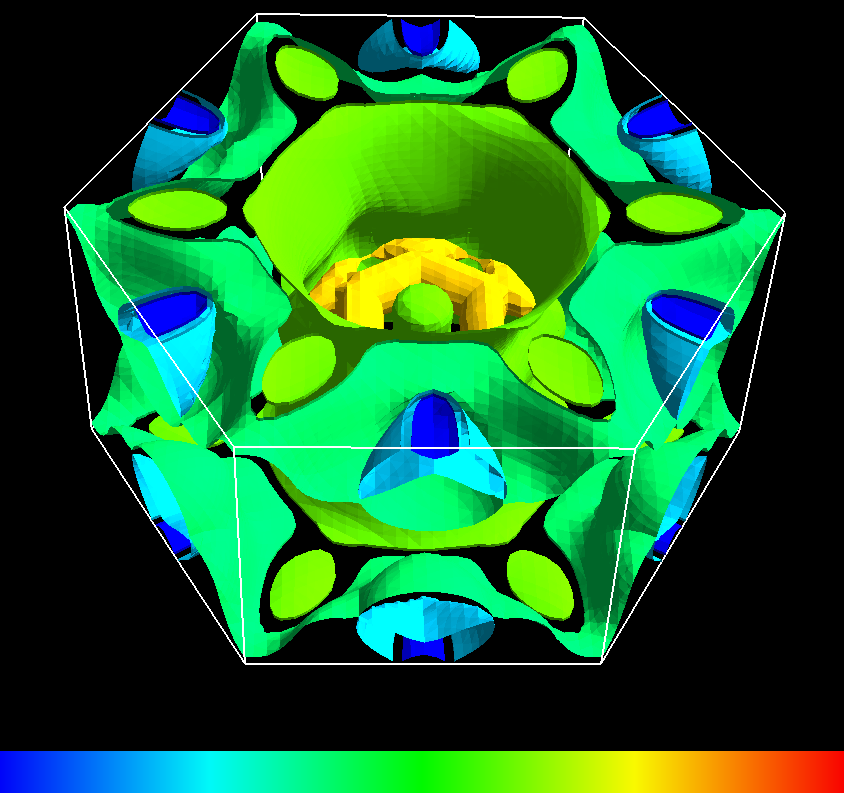


**b**

α = 1.00

~~
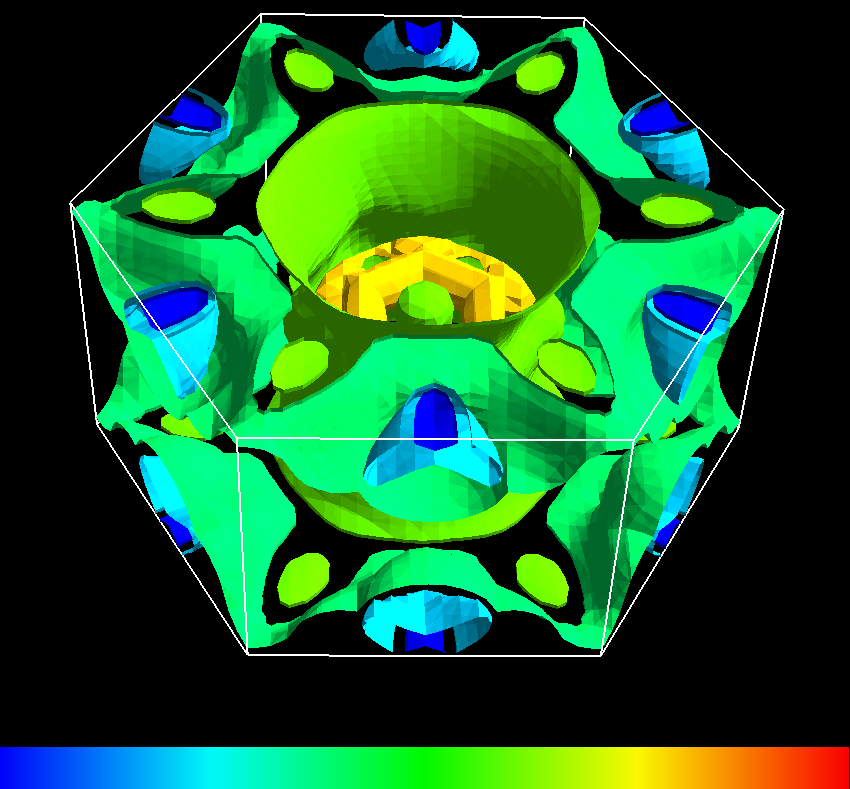
~~

**c**

α = 1.01

**Fig. S5**

**Calculated Fermi Surface for Re with different lattice parameters.** To this end, a lattice scaling factor α is introduced; the α =1.00 case (**b**) means no scaling result [with experimental structure (*a* = 2.758 Å, *c* = 4.447 Å)] and the α =0.99 (**a**) and 1.01 (**c**) cases indicate that the lattice parameters are uniformly scaled-down and -up, respectively. The Fermi surface is calculated with “FermiSurfer” [35].

**Table S1**

**Physical properties of a series of filed Re specimens.**

The effect of filing by sandpaper of #1000 is saturated at around 1.5×10^3^ times filing. Further change was brought about by changing sandpaper from #1000 to #240. More prominent change appears by changing sandpaper into #60.

| Sample No. | Sandpaper number and number of rotation style of filing at each step/ total number | *T*_c_ [K] | mass  *m* [mg] | thickness  *t* [mm] | density  *n* [g/cm^3^] |
| --- | --- | --- | --- | --- | --- |
| #0 | After pressing | - | 67.3 | 0.40 | - |
| #1 | After light filing for flattening | 2.63 | 64.9 | 0.25 | 20.7 |
| #2 | #1000  1460 / 1460 | 3.01 | 61.0 | 0.24 | 20.2 |
| #3 | #1000  +1300 / 2760 | 2.92 | 59.5 | 0.22 | 21.5 |
| #4 | #1000  +3000 / 5760 | 3.04 | 53.1 | 0.20 | 21.1 |
| #5 | #1000  +2500 / 8260 | 2.83 | 50.1 | 0.20 | 19.9 |
| #6 | #1000  +3400 / 11660 | 2.99 | 46.4 | 0.17 | 21.7 |
| #7 | #1000  +3200 / 14860 | 2.91 | 38.7 | 0.15 | 20.5 |
| #8 | #1000  +4000 / 18860 | 3.05 | 33.6 | 0.13 | 20.6 |
| #9 | #240  +2600/21460 | 3.12 | 27.3 | 0.12 | 18.1 |
| #10 | #240  +4000/25460 | 3.18 | 18.9 | 0.09 | 16.7 |
| #11 | #60  +500 / 25960 | 3.28 | 17.0 | - | - |

**Table S2**

**Structural parameters such as the crystalline size *D*, crystalline strain** ***ε*, lattice constants *a*, and *c* in strained Re.** The corresponding XRD patterns are presented in Fig. S2. The measurement of the as-received sample has been conducted twice, and the data for as-received (2) are presented in Fig. 3.

| Stress*, P* | *N* of HPT | *D* [nm] | *ε* | *a* [Å] | *c* [Å] |
| --- | --- | --- | --- | --- | --- |
| as-received (1) |  | 185.2  ±40.2 | 0.090  ±0.015 | 2.7601  ±0.0007 | 4.4595  ±0.0009 |
| as-received (2) |  | 149.3  ±44.4 | 0.084  ±0.017 | 2.7604  ±0.0008 | 4.4594  ±0.0010 |
| 24 GPa | 0 | 55.7  ±45.5 | 0.29  ±0.11 | 2.7620  ±0.0010 | 4.4600  ±0.0013 |
| 24 GPa | 1 | 42.4  ±27.2 | 0.56  ±0.12 | 2.7618  ±0.0012 | 4.4610  ±0.0002 |
| 24 GPa | 10 | 32.6  ±17.6 | 0.48  ±0.20 | 2.7640  ±0.0040 | 4.4651  ±0.0030 |
| filed (#11) |  | 13.7  ±2.4 | 0.25  ±0.08 | 2.7649  ±0.0010 | 4.4633  ±0.0019 |
